# Multifunctionalized Conductive Polymers for Self-Healing Silicon Anodes in Li-Ion Batteries

**DOI:** 10.1021/acsomega.5c04052

**Published:** 2025-07-24

**Authors:** Neslihan Yuca, Omer Suat Taskin, Emre Guney, Javier García-Alonso, David Maestre, Bianchi Méndez

**Affiliations:** † Enwair Energy Technologies Corporation, Kağıthane, Istanbul 34415, Turkey; ‡ Institute of Energy, 52971 Istanbul Technical University, Istanbul 34469, Turkey; § Department of Chemical Oceanography, Institute of Marine Science and Management, 37516 Istanbul University, Istanbul 34134, Turkey; ∥ Departamento de Física de Materiales, Facultad de CC. Físicas, 16734 Universidad Complutense de Madrid, Madrid 28040, Spain

## Abstract

Silicon is a very promising material for lithium-ion batteries (LIBs) due to its high theoretical capacity (3579 mAh/g). However, the volumetric expansion (300%) of silicon during lithiation led to pulverization of the electrode and rapid capacity fading. Self-healing (SH) materials are thought of as a solution for the degradation of active materials, enabling higher capacity retention. Here, we synthesized and integrated an autonomous self-healing poly­(aniline-*co*-3-aminophenylboronic acid)/PVA composite (SHC) as a binder in a Si anode electrode for LIBs. The synthesized SHC was investigated by Fourier transform infrared (FTIR) spectroscopy, thermogravimetric analysis, and elongation and conductivity tests. Si anodes were prepared with SHC and a PVP cobinder. In addition, Si anodes were prepared separately with PVDF and the CMC-SBR binder as control electrodes. The electrodes were electrochemically characterized by electrochemical impedance spectroscopy, cyclic voltammetry, and galvanostatic charge/discharge tests. The conductive SHC binder was successfully integrated into the Si anode, and a capacity of over 1700 mAh/g was obtained after 100 cycles at C/10, and 650 mAh/g was obtained after 200 cycles at C/2.

## Graphical abstract

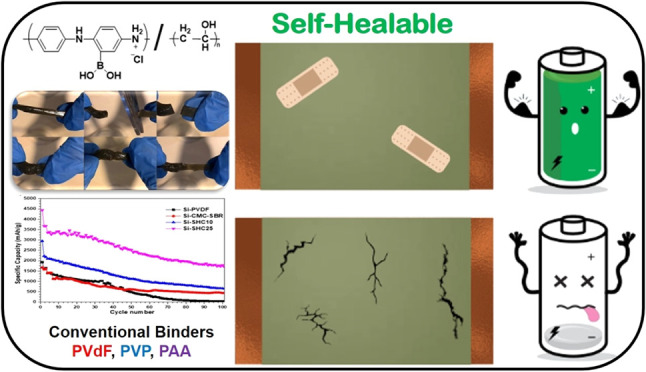

## 1 Introduction

Today, it is possible to obtain a synthetic material that can regain its structural integrity after any damage.^1^ Polymers, metals, ceramics, and composites can be used as self-healing materials. Whether healing is autonomous or not is classified according to its dependence on any external stimuli, such as temperature, ultraviolet (UV) radiation, or external intervention.^2,3^ Fractures and other damages in materials start microscopically because the fracture energy cannot be effectively distributed in the structure, and the fracture grows and spreads throughout the material. The synthesis of polymers, in which damage can be easily controlled or healed, has become very important due to their intense use in aircraft, cars, ships, defense, and construction sectors.^4–6^ Materials with self-healing ability can partially or completely return to their original state after damage to their structures. Ideal self-healing materials, on the other hand, can suddenly and completely return to their original properties as soon as the cause of damage is eliminated. Self-healing can occur in polymers, sometimes spontaneously and sometimes by external influences. Self-healing can be achieved in polymers in many ways, such as microencapsulation, hydrogen bond interactions, and disulfide bonds.^7,8^

Embedded healing agents are one of the well-known self-healing materials that have built-in microcapsules (small embedded pockets) filled with an adhesive-like chemical that can repair the damage. If the material cracks inside, the capsules open, the repair material “ejects,” and the crack closes. It works similarly to an adhesive (glue) called epoxy, which is supplied in the form of two liquid polymers in separate containers (usually two syringes). When it mixes liquids, a chemical reaction occurs, and a strong adhesive (a copolymer) is formed. Self-healing materials can be embedded in capsules in various ways. The most straightforward approach for capsules is to release an adhesive that fills the crack and binds the material together.^9,10^

Examining the self-healing behavior provided by the inclusion of reversible bonds in the polymer matrix is one of the new approaches. Healing is initiated by bringing the cut surfaces together or by performing a chain reaction with the solvent. One of the recent ideas regarding self-healing is the bringing together of the broken or cut surfaces of rubber-like polymer, formed by hydrogen bonds. Here, self-healing begins with the heating of the material, and the fractured hydrogen bonds are reformed.

Polymers endowed with self-healing properties utilize reversible hydrogen (H) bonding mechanisms to restore their structural integrity following damage. This self-repair process hinges upon secondary interactions, wherein noncovalent H bonds undergo dynamic dissociation and reassociation in response to external stimuli, such as heat, pressure, or moisture. Upon the occurrence of a crack, the H bonds at the damaged interface may dissociate; however, they can subsequently reassociate due to their inherent reversibility. The effectiveness of this self-healing capability is further enhanced by the availability of multiple hydrogen bonding sites, such as ureidopyrimidinone (UPy) or carboxyl groups, which facilitate supramolecular networking. Such interactions enable the polymer to autonomously effect repair by diffusing and realigning molecular chains, thereby facilitating bond reformation and mechanical recovery.^10–14^

The efficiency of the healing process is influenced by several factors, including bond strength, polymer mobility, and environmental conditions. Stronger hydrogen bonds contribute to more robust healing; nonetheless, they may impede dynamic rearrangement. Conversely, weaker bonds enhance the polymer’s adaptability. This delicate balance promotes the applicability of self-healing polymers in domains such as coatings, elastomers, and structural materials, where the ability to recover from damage significantly extends the lifespan of the materials involved.^15–18^

Si anodes have become popular in battery research due to their high theoretical capacity (3579 mAh/g) and storage capacity of a single silicon atom, which is 4.4 Li atoms. This phenomenon increases the volumetric energy density of the battery. However, Si atoms undergo too much volumetric change (300% expansion) during Li intercalation/deintercalation (charge/discharge), which leads to an unstable solid–electrolyte interface (SEI) and pulverization of Si particles.^19–22^ Also, silicon is a semiconductor, and thus, high-purity Si has a very low electrical conductivity at room temperature (<10^–5^ S/cm). These disadvantages cause rapid capacity fading.^23,24^ Traditionally, binders have been used as a soft matrix backbone that allows volume expansion of the anode or cathode while preserving its morphology.^25–29^ These polymeric binders are used to ensure the adhesion of other materials to each other and to the current collector foil. Carboxymethyl cellulose (CMC), alginates, poly­(acrylic acid) (PAA), poly­(vinylidene fluoride) (PVdF), poly­(vinylpyrrolidone) (PVP), and poly­(vinyl alcohol) (PVA), which interact strongly with particles, are among the commonly used polymeric binders.^30–37^ Polymers with functional groups such as hydroxyl or carboxylate exhibit strong hydrogen bonding to stabilize the Si anode by hydrogen bonding to silicon hydroxide (SiOH) on the Si surface.^38^ Alginate, carboxymethyl cellulose (CMC), and poly­(acrylic acid) (PAA) were the first applied polymers to overcome Si anode instability.^39^ Poly­(acrylic acid) (PAA) is widely used as a polymer binder for high-capacity silicon (Si) anodes in Li-ion batteries.^36^ When the carboxyl (single-bond CO_2_H) groups of PAA are used, it facilitates the lamination process, especially for large-scale production. PVDF is one of the most famous polymer binders used for electrodes. PVDF is commercially used as a cathode and anode material. Although PVDF has electrochemical and thermal stability and good adhesion between the current collector and active materials, it does not have sufficient mechanical integrity to inhibit anode pulverization during the expansion of Si nanoparticles.^37–39^ Smart materials, such as self-healing polymers, have been investigated to overcome these disadvantages. It has been observed that self-healing polymers have a positive effect on hindering the pulverization of Si nanoparticles and cracking in the electrodes. Self-healing polymers are examined under two main headings: irreversible and reversible polymers due to their self-healing mechanism.^40^ Irreversible self-healing polymers, such as encapsulated self-healing polymers, show material recovery when the capsule is broken by external damage. This capsule breakage allows the polymer to fill the cracks formed and cure the material once. Reversible self-healing polymers show recovery of the material several times. This type of self-healing polymer, especially autonomous self-healing polymers, showed remarkable results in Si anode stability over long cycles.^45^ Autonomous self-healing polymers have mobile weak bonds that allow recombination in the polymer matrix with hydrogen bonding, S–S bond, or weak van der Waals bonds when the bonds are broken.^42^ Some attempts at SH polymer binders containing PANI or borax structures for the Si anode are reviewed in Table 1.

**Table 1:** Si Anode Studies of the Investigated Self-Healing Binders

material	self-healing method	electrochemical performance	refs
silicon–poly(ethleyene glycol)–boronic cross-linked guar	boronic cross-linker bonds and hydrogen bonding	1000 mAh/g after 300 cycles at 0.2C, 87.3% ICE, ∼0.7 mg cm^–2^	^43^
silicon-esterified poly(acrylic acid)	borate ester of the healing mechanism	1500 mAh/g after 500 cycles at 1 A g^–1^, 82.1% ICE, 0.75 mg cm^–2^	^44^
silicon–polyaniline–sulfonated graphene nanosheet	cross-linker hydrogen bonding	550 mAh/g after 5000 cycles at 6.0 A g^–1^, 70% ICE, 0.2–0.3 mg cm^–2^	^45^
silicon–polyaniline	enhancement of hydrogen bonding	386.4 mAh/g after 200 cycles at 0.1C, 77.56% ICE, 0.455 mg cm^–2^	^46^
silicon–citric acid/poly(acrylic acid)	enhancement of hydrogen bonding	3150 mAh/g after 300 cycles at 0.1C 89.5% ICE, 0.6 mg cm^–2^	^47^
silicon–poly(aniline-*co*-3-aminophenylboronic acid)/PVA	hydrogen bonding and conductive polymer	1706 mAh/g after 100 cycles at 0.2C, 79.2% ICE, 0.77 mg cm^–2^ 677 mAh/g after 200 cycles at 1C	this study

Conventional binders such as PVDF and CMC offer structural adhesion but are inadequate in preventing crack propagation and ensuring conductivity, while extrinsic self-healing systems (e.g., capsule-based) provide localized repair at the expense of conductivity and necessitate external triggers; in contrast, intrinsic self-healing binders enhance autonomy through dynamic bonding yet often compromise conductivity, whereas self-healing conductive (SHC) binders effectively integrate autonomous repair mechanisms with inherent conductivity, thereby addressing the shortcomings of both conventional and self-healing systems, although challenges persist in optimizing the balance between healing kinetics and conductivity for industrial scalability^43–50^

Wang et al. reported the first example of a PAA–PVA hydrogel based on a borate ester bond in an energy storage application.^47^ The PAA part provides good flexibility and high ionic conductivity to the PVA hydrogel, while the borate ester bonds between the borax and PVA chains can fully restore the mechanical performance of the gel at room temperature to its dynamics. Li et al. obtained high energy density and high capacitance in flexible solid-state supercapacitor applications by using intermolecular interactions of polyaniline and poly­(vinyl alcohol), which show self-healing properties owing to dynamic borate bonding.^48^ Ubiquitously curable battery electrode materials are integrated into polymeric networks cross-linked by dynamic borate ester bonding, restoring the material’s mechanical properties and lithium storage performance without an external stimulus.^49^

The use of the boronic cross-linker guar (BC-g) as a binder in the Si electrode, together with the formation of a covalent bond from the BC, provides the self-healing ability of poly­(ethylene oxide) (PEO) and boronic ester, abundant hydroxyl groups, and rapid lithium-ion transport via PEO. The mechanical properties of the BC-g polymer are significantly promoted toward spontaneous room temperature gelation (or close cross-linking between alcohol and boronic acid).^50,76^ In the study of Jung et al., the electrode produced with poly­(acrylic acid) consisting of −COOH groups as a binder for the ester bond and modified silicon with −OH groups on the surface offers a high capacity of 1500 mAh/g after 500 cycles, exhibiting excellent high-speed capability and long-cycle stability.^51^ Wu et al. reported that the three-dimensional polyaniline framework, as well as the coherent conductive coating surrounding each Si electrode, can be cycled up to 5000 times without significant loss of capacity.^45^ Mi et al. reported that, in contrast to the pristine Si, Si-SA, and Si-PANI composite electrodes, the Si-SA-PANI composite electrode showed a superior rate capacity, longer cycle life, and higher specific capacity.^52^ This type of sulfonated boronic acid mechanism is also expected to be a good reference for the synthesis of anode materials damaged by significant volume changes in cycles. Conductive polymers such as poly­(3,4-ethylenedioxythiophene) polystyrenesulfonate (PEDOT:PSS),^53,54^ polypoyrrole (PPy),^55^ and polyaniline (PANI)^56^ are also used to overcome the conductivity drawbacks of Si NPs in electrodes.

Many studies have proven that conductive polymers are used as binders or conductive material additives to contribute to the capacity of electrodes with their unique electron conduction properties.^57–61^ Intrinsic self-healing polymers are one of the most investigated autonomous self-healing polymers for stable Si nanoparticle-based anodes.^41^ It is crucial that the binder has a mobile polymer backbone and can self-heal after any deformation of the polymer. Here, we synthesized a multifunctional poly­(aniline-*co*-3-aminophenylboronic acid)/PVA composite binder: first, as an intrinsic self-healing polymer with hydrogen bonding mechanisms (Figure 1); second, as an autonomous healing system to maintain the integration of a Si anode as a binder during the Li-ion cell working process. Third, it is planned to reduce the impedance of the electrodes prepared with the conductive polymer features of the polyaniline backbone of the SHC binder. Fourth, the presence of both aromatic groups in the main chain polymer and aliphatic groups in the counter polymer increases the durability and mechanical strength of the structure. Detailed information regarding the polymer structure and experiments is provided in Figure S1. A high-performance Si anode electrode with stability during performance testing was achieved by using a synthetic polymer binder with both self-healing and conductive properties.

**Figure 1:**
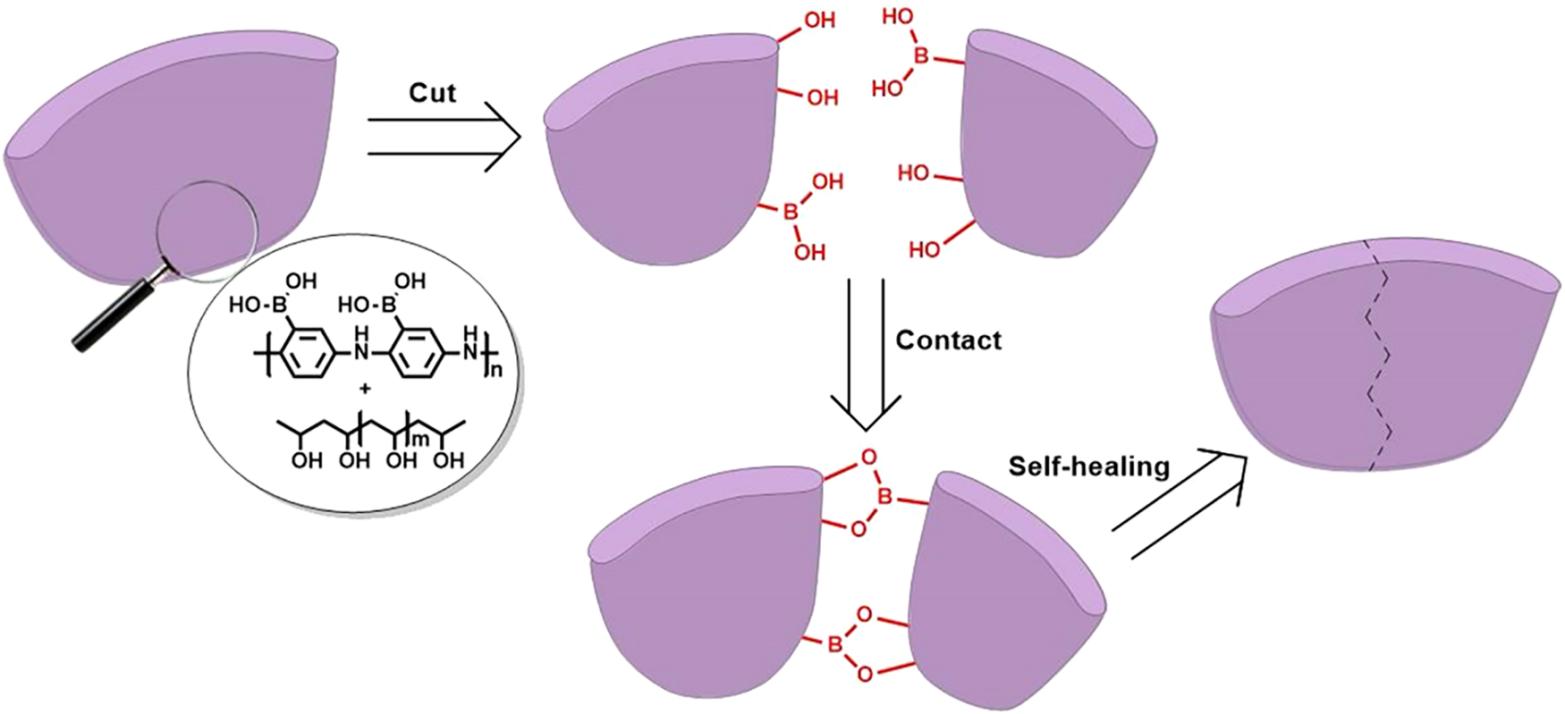
Schematic representation of the self-healing mechanism of the SHC binder.

## 2 Results and Discussion

The SHC gel exhibited twice the elongation strength of its original shape without any separation under manual tensile testing (Figure 2a). The material has the ability to elongate due to its molecular structure, and when it comes together again after cracking or rupture, it regenerates infinitely via H bonds, allowing the damaged structure to be repaired. This healing mechanism can occur within seconds due to the effect of hydrophobic interactions via H-bond chemistry. Figure 2b shows the self-healing steps of the SHC. The synthesized gel healed on its own in less than 1 min after being cut with scissors. This process is proof of the self-healing ability achieved by the SHC.

**Figure 2:**
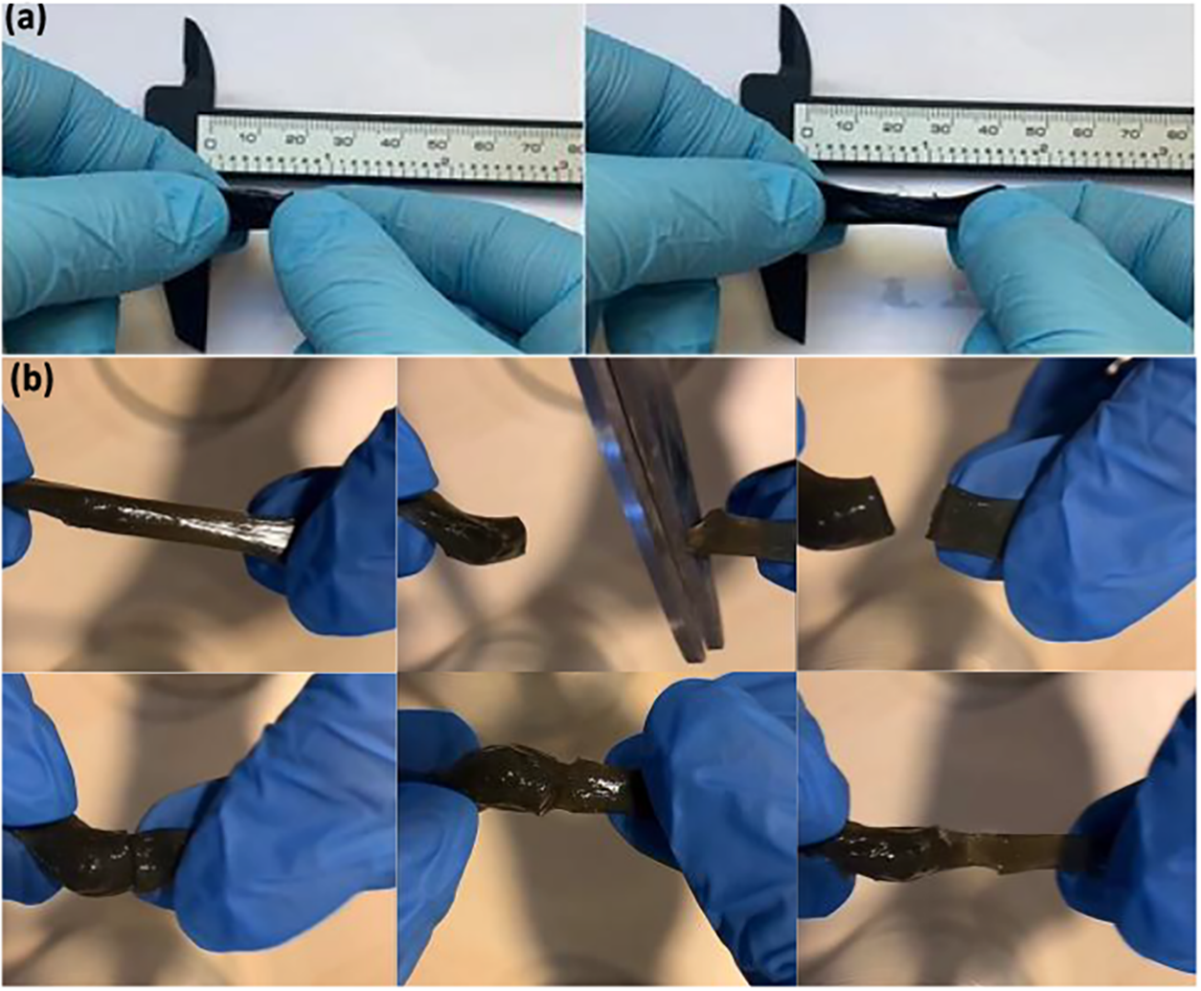
(a) Elongation test of poly­(aniline-*co*-3-aminophenylboronic acid)/PVA and (b) self-healing test of poly­(aniline-*co*-3-aminophenylboronic acid)/PVA.

The SHC polymer was chemically characterized to analyze its conductivity and to have a better understanding in terms of the thermal and structural behavior of the healing mechanism. The polyconjugated nature of the main chain polymer gives higher conductivity due to the delocalization of π-bonds in anilines.

Aromatic aniline groups around 1400 cm^–1^, which are conductive to the binding system, and C–O groups of the vinyl alcohol repeating units at 1100 cm^–1^ can be clearly seen in the corresponding FTIR spectrum shown in Figure 3a. The N–H bonds of the aniline are readily seen at wavenumbers of 2800–2900 cm^–1^. In the FTIR spectrum, two types of hydrogen bonding can be considered: (1) intermolecular hydrogen bonding between two PANI (boronic acid) hydroxyl groups and encounter poly­(vinyl alcohol) lateral groups, and (2) intramolecular hydrogen bonding between boronic acid hydroxyl units and nitrogen atoms on the aniline groups. These hydrogen bonding attractions can easily be detected in the infrared (IR) spectrum of the final polymer as broadening of the OH stretching bands at 3400 cm^–1^ and the shift in this region to higher wavenumbers, proving that H-bonds are dynamically ascendant in the healing mechanism (Figure 3b).

**Figure 3:**
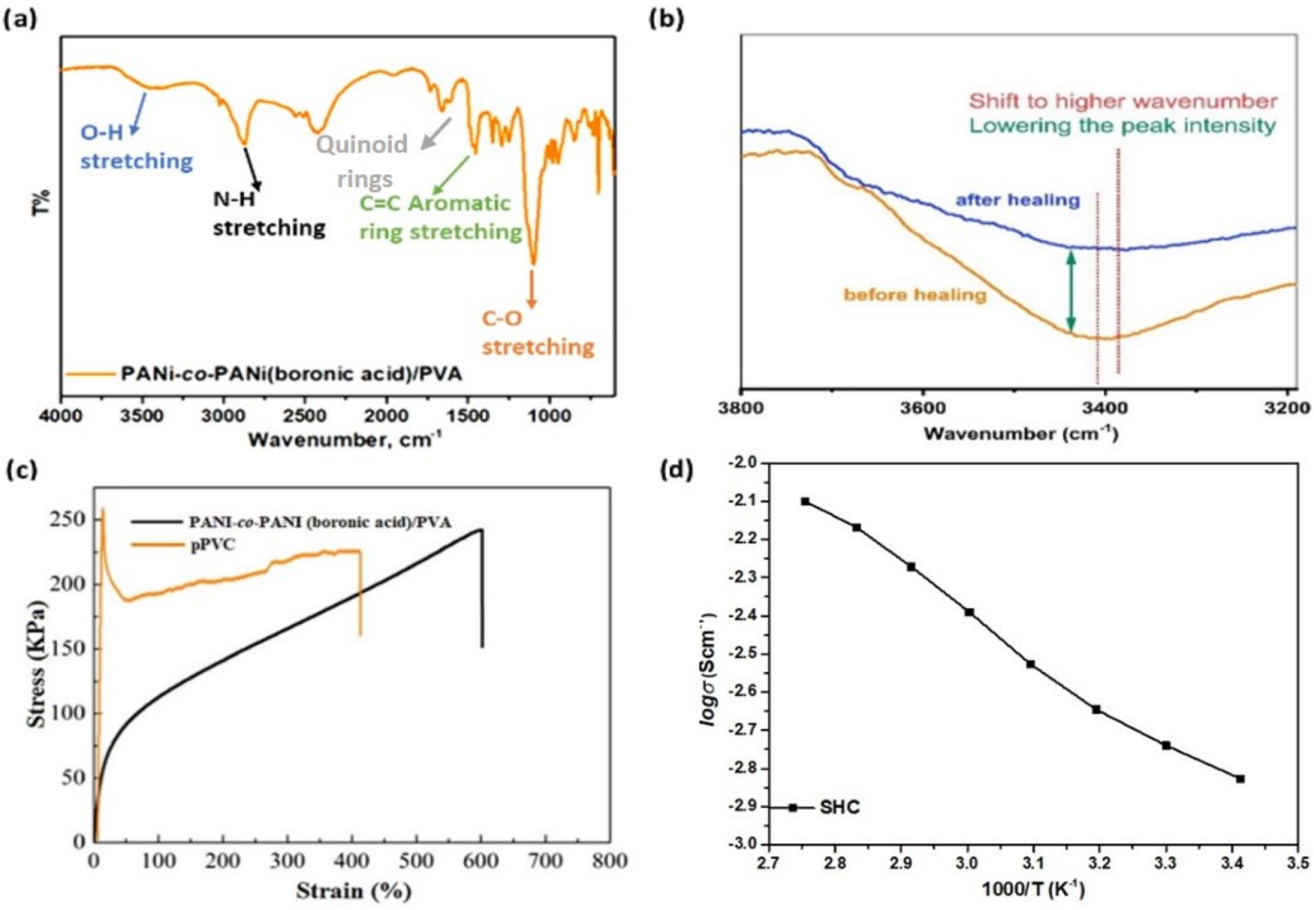
(a) FTIR spectrum of the synthesized PANI-*co*-PANI (boronic acid)/PVA (SHP); (b) shift of hydroxyl peaks in FTIR spectra after healing of PANI-*co*-PANI (boronic acid)/PVA; (c) tensile stress–strain curve of PANI-*co*-PANI­(boronic acid)/PVA, and (d) conductivity measurement of the SH polymer.

The synthesized self-healing composite polymer formed a hydrogel with the help of hydrogen bonds between poly­(3-aminophenylboronic acid–aniline) and PVA, which provided mechanical strength to the SHC polymer (Figure 3c). PVA as a counter polymer is expected to enhance the self-healing capability by providing OH– units. To support the structural and morphological analyses of the polymer, Raman and scanning electron microscopy (SEM) analyses were conducted, as shown in Figures S2 and S3. The proton conductivity of the SHC was investigated under anhydrous conditions at different temperatures. According to the conductivity measurement of the SHC polymer shown in Figure 3d, it was found that the conductivities were 1.7 mS/cm at 20 °C and 7.7 mS/cm at 90 °C.

Thermogravimetric analysis was performed to understand the thermal behavior of the composite polymer (SHC). As seen in Figure S4, the weight loss was measured up to an ambient temperature of 600 °C in the TGA profile. There are three main weight loss stages of the structure, according to the TGA curve of the final polymer composite. While the initial weight loss is due to the loss of water and volatile solvents at about 110 °C, the major weight loss is due to the thermo-oxidative decomposition of polyaniline and the counter polymer PVA in the structure, resulting in *N*-phenylaniline, methane, acetylene, carbazole, and other decomposition products. A high rate of weight loss occurred between 220 and 410 °C.

Despite some characterization methods providing a combination of knowledge about the self-healing polymer structure, it is required to show the healing mechanism that is working outside the cell. To provide this experience, a current flow test system was built, and the illustration in Figure 4 shows a current flow test conducted on a self-healing polymer that was intentionally cut and subsequently allowed to heal. The self-healing polymer, characterized by its ability to repair damage autonomously, is shown to restore its electrical conductivity postrepair. This proves that the SHP has successfully reconnected the electrical pathways, as evidenced by the restoration of the current flow through the previously damaged or cut area. This property is crucial for applications in flexible electronics, where maintaining the electrical integrity despite mechanical damage is essential. The successful restoration of the current flow signifies the material’s potential for prolonged durability and reliability in practical applications.

**Figure 4:**
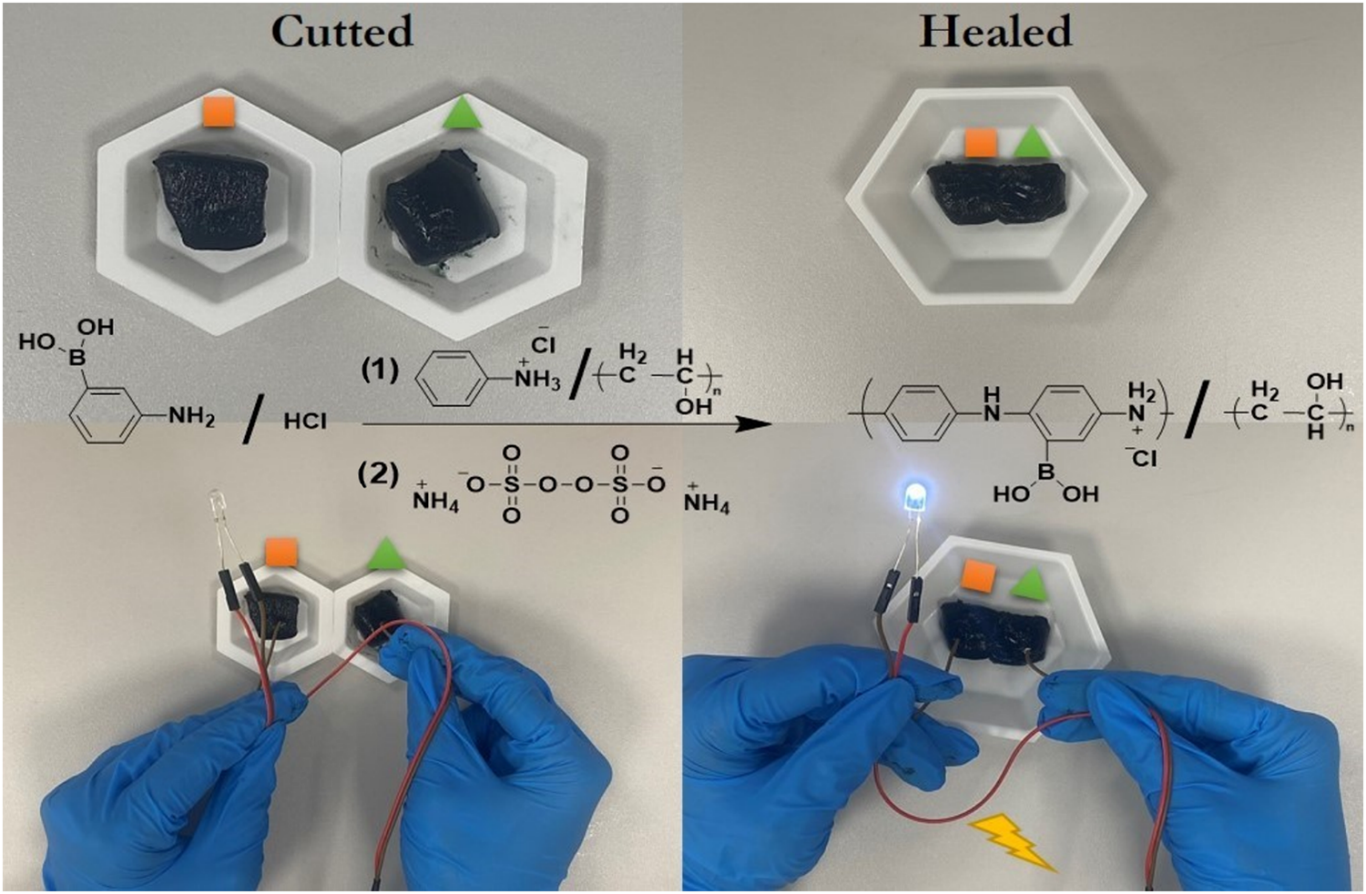
Illustration of the current flow test for cut and healed SHP.

Figure 5 shows the X-ray photoelectron spectroscopy (XPS) spectra of the pristine (uncycled) Si anode electrode prepared with SHC25, SHC10, CMC-SBR, and PVDF binders as A, B, C, and D, respectively. The electrode compositions are summarized in Table 2. Additionally, the XPS measurements of the electrodes after formation at C/25 for 4 cycles and at C/10 for 4 cycles are shown in Figure S5. Pristine electrodes prepared using different binders showed almost the same Si 2p binding energy, except for the CMC/SBR binder. In the XPS spectrum of Si, the 2p binding energy at around 99.5 eV is assigned to bulk silicon, and the other peaks correspond to SiO_2_ at 103.5 eV.^60–63^ The reason that the SiO_2_ of the Si-CMC/SBR electrode is more than Si is related to the free hydroxyl group on the CMC polymer. These hydroxyl groups interact with Si NPs and generate SiO_2_ at the electrode.^60^ However, it was observed that the Si electrodes containing the SHC polymer binder (Figure S5B) did not show any peak at the former binding energy level of the Si 2p state for bulk Si or SiO_2_ after the formation cycles of the electrodes. This is due to the self-healing nature of the SHC polymer as well as the formation of SEI, which envelops the Si active materials and provides them with long-term cyclicity without excessive loss of capacity.^61,62^ It is also observed that Si-CMC/SBR and Si-PVDF electrodes exhibit Si 2p peaks at around 99.5 and 103.5 eV, corresponding to bulk Si and SiO_2_, respectively. From these measurements, it is also evident that the SHC polymer provides high levels of protection for Si anodes from the start. The O 1s spectrum of the electrodes observed by XPS (Figure 5A–C) showed signals of O environments in native oxide SiO_2_/C–O and C=O species at 532.5 and 531 eV, respectively. The C 1s results of XPS analysis indicate carbon bonds in the binders.^66^ In Figure 5A,B, 4 different peaks were observed, which correspond to a binding energy of 284.5 eV, assigned to hydrocarbons such as C–H and C–C. In addition to the hydrocarbon peaks, the peak at 286 eV corresponds to the −CH_2_–N bonds in the PVP cobinder.^67,68^

**Table 2:** Summary of the Electrode Compositions

sample	active material (wt %)	SHC binder (wt %)	Cobinder (wt %)	conductive additive (wt %)
Si-SHC10	70	10	8	12
Si-SHC25	55	25	8	12
Si-PVDF	80		10	10
Si-CMC/SBR	80		10	10

**Figure 5:**
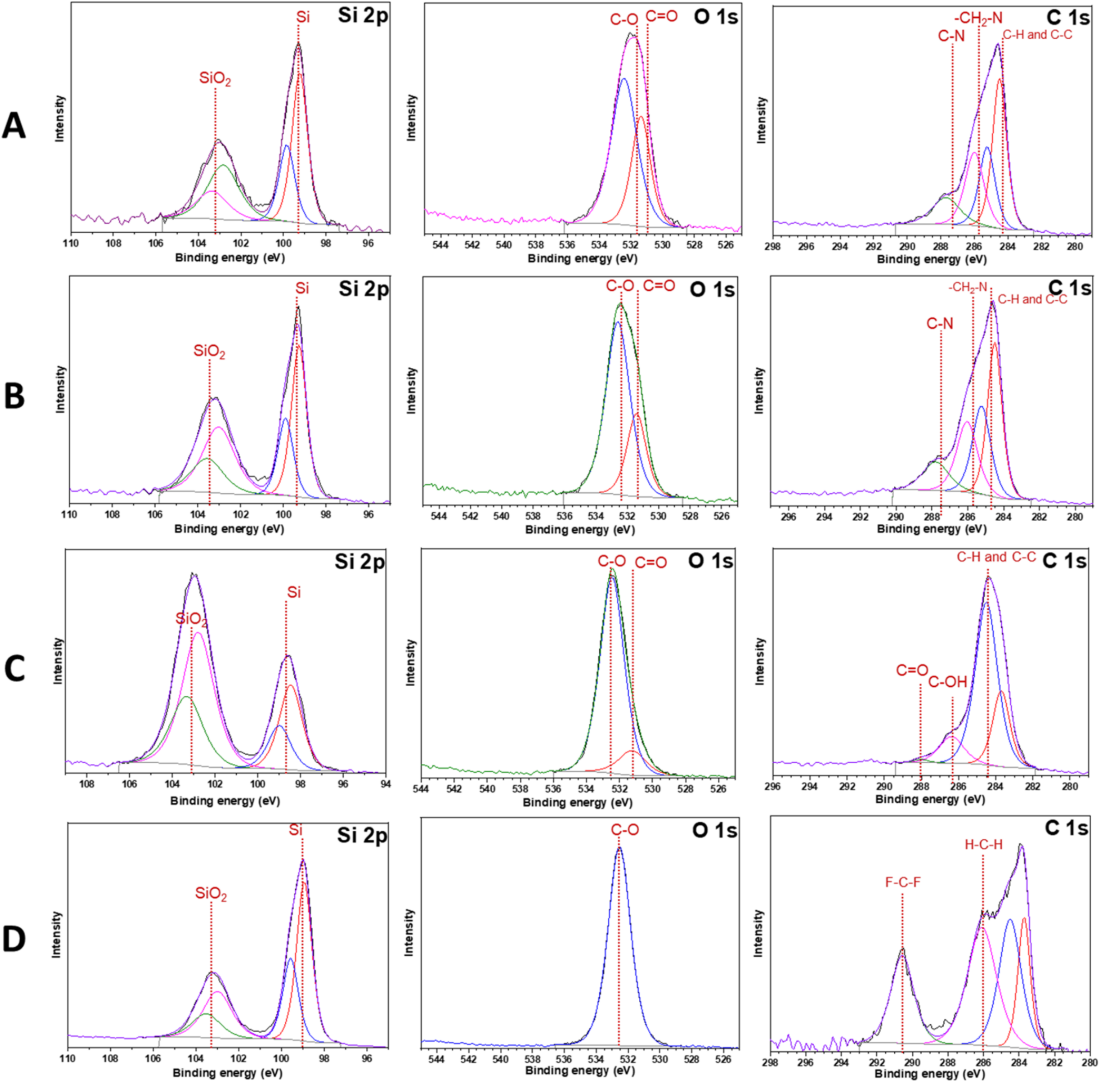
XPS measurements of the (A) Si-SHC25 electrode, (B) Si-SHC10 electrode, (C) Si-CMC/SBR electrode, and (D) Si-PVDF electrode.

Finally, C–N/C=N and C–N+/C=N+ bonds were assigned to binding energies of 285.23 and 287.7 eV, respectively.^35^ Similar to Figure 5A,B, in Figure 5C,D, a binding energy of 284.5 eV was assigned to hydrocarbons. Unlike results A and B, peaks were observed at 286.4 and 288 eV, corresponding to the C–OH and C=O bonds of the CMC binder, as shown in Figure 5C, respectively.^64,69^ As shown in Figure 5D, a peak was observed around 290 eV in the PVDF binder, which was attributed to the F–C–F bond.^70,74^

Conductive polymers are used in battery electrodes to improve electrical conductivity, which also provides better specific capacity.^55^ Since pristine silicon in nature has low electrical conductivity and volume change during the lithiation/delithiation process, SHC was used as a binder to enhance Si anode performance with conductive features and ensure the durability of the Si anode by showing a characteristic self-healing mechanism.^59^ The Si anode was prepared using different commercial binders and with an SHC binder to compare each electrode’s performance.

Electrochemical impedance spectroscopy (EIS) measurements (Figure 6a) showed that the Si-SHC25 electrode had a lower impedance of 30.56 ohm compared to Si-PVDF and Si-CMC-SBR (87.76 and 100.6 ohm, respectively), proving its electrical conductivity. The equivalent circuit shown in the inset of Figure 6a was applied to each impedance measurement to determine the impedance of each electrode. *R*_b_ represents the bulk resistance of the half-cell, *R*_int_ represents the formed SEI of the anode electrode, *R*_ct_ represents the charge transfer resistance of the electrode, and C9 and C10 represent the capacitive effect between the SEI electrolyte and electrode–electrolyte, respectively.

**Figure 6:**
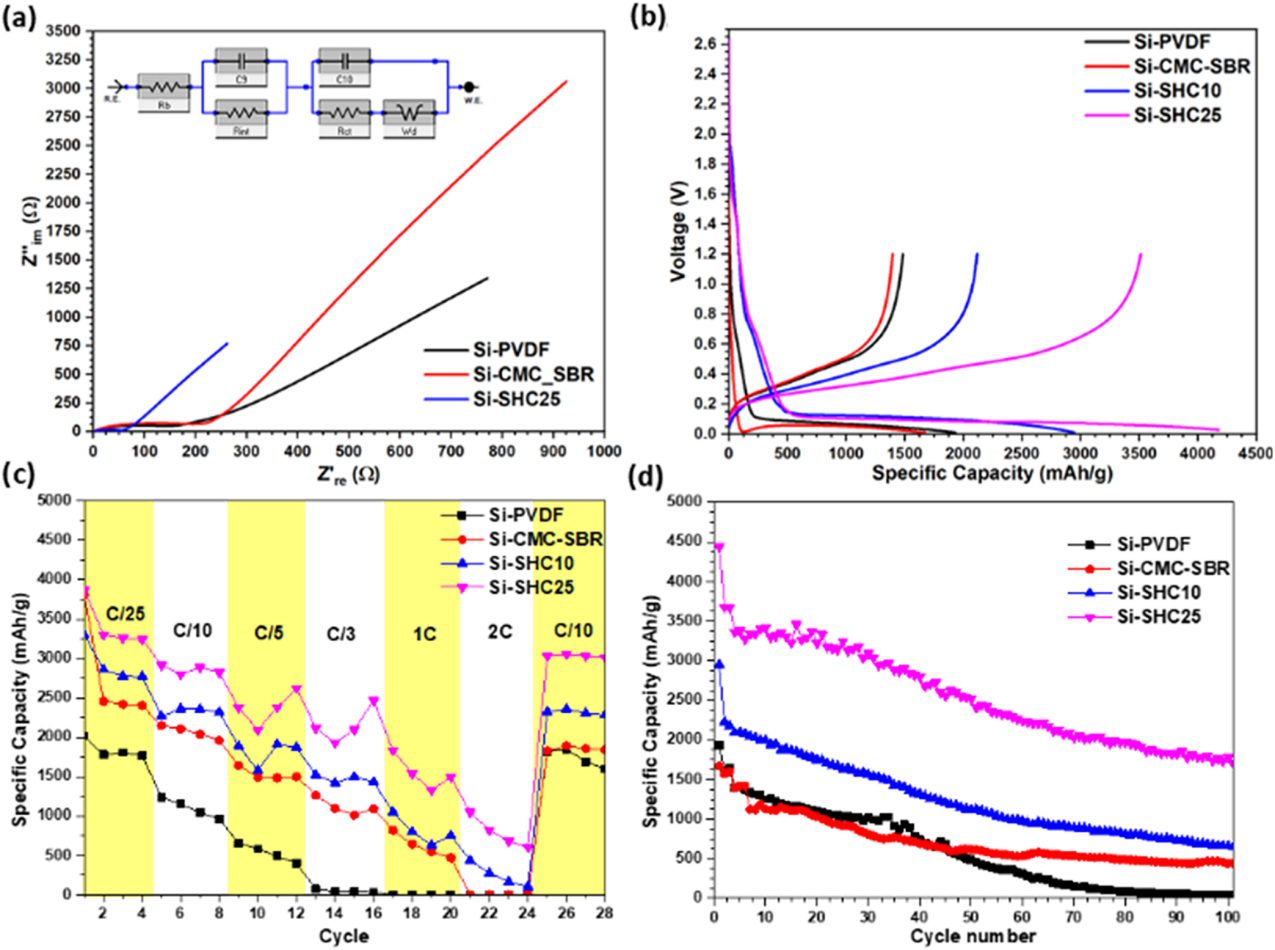
Comparison of (a) EIS results with the equivalent circuit shown as an inset, (b) capacity–voltage graphs of electrodes at first cycles, (c) C-rate results, and (d) galvanostatic charge/discharge test results.

Figure 6b shows the first cycle voltage profile of the electrodes. The plateau at <0.15 V in the first discharge curve can be attributed to the formation of amorphous LixSiy as the insertion of lithium. When Si-SHC25 was used, higher lithiation (4125 mAh/g) and delithiation (3500 mAh/g) capacities were observed, while the electrodes with PVDF or CMC as binder showed lower capacities. Similarly, Si-SHC25 has a higher capacity than the others at different C rates, which can be attributed to the continuous conductive network in the whole electrode. The capacity–voltage curves after several representative cycles are shown in Figure S6 for each sample. Figure 6c shows that the Si-SHC25 electrodes stably continue to show the highest capacity at different charge/discharge rates and operate at C/10 after performing different C rates. The Si anode suffered from a galvanostatic charge/discharge test and had a sharp reduction in capacity because of pulverization during the cycle. Degradation of Si was suppressed by the SHC polymer with the Si-SHC25 composition compared to other binders. Also, the composition of the SHC affects the electrode capacity and capacity retention, as shown in Figure 6d. By increasing the quantity of the SHC binder from 10 to 25%, the specific capacity of the Si anode increased from 2945 to 4125 mAh/g. These results exceed those of the PVDF and CMC-SBR-based Si anode electrodes, which had specific capacities of 1931 and 1671 mAh/g, respectively. Si-PVDF did not show any capacity after 100 cycles at a C/10 charge/discharge rate. Although Si-CMC-SBR and Si-SHC10 showed almost the same capacity at the end of 100 cycles, the Si-SHC25 electrode showed a much better capacity than the others, with over 1706 mAh/g. More detailed electrochemical investigations have been applied to Si-SHC25 anode electrodes to gain a deeper understanding of the multifunctional self-healing binders. The Coulombic efficiency of the electrode during the galvanostatic charge–discharge cycles is shown in Figure S7. It is observed that the Si-SHC25 electrode showed 79.2% initial Coulombic efficiency (ICE), while the Si-SHC10, Si-PVDF, and Si-CMC/SBR electrodes showed ICE values of 72, 83.4, and 76.9%, respectively.

More detailed electrochemical investigations have been applied to Si-SHC25 anode electrodes to achieve a deeper understanding of the multifunctional self-healing binder. The healing ability of the self-healing polymer performed well even at high C rates (Figure 7a). The fast-charging ability of Si-SHC25 is measured based on the data obtained from the cycling test at C/10. Therefore, the Si-SHC25 electrodes were assembled as half-cells again, and the cells were cycled under C/2 and 1C test conditions after the formation process. The formation process is a slow condition test in which current is applied to the assembled cells according to the specific capacity of the anodes at C/25 and C/10. The Si-SHC25-C/2 and Si-SHC25–1C electrodes had specific capacities of 677 and 615 mAh/g, respectively, after 200 cycles. Although the specific capacity of the electrodes continued to decrease until 200 cycles, the SHC maintained the integrity of the electrodes, performing at high C rates. Figures S8 and S9 show the cycling performance of the electrodes with PVDF and CMC/SBR at C/2. It is well known that material degradation and SEI formation increase due to electrolyte decomposition at high C rates.^72^

**Figure 7:**
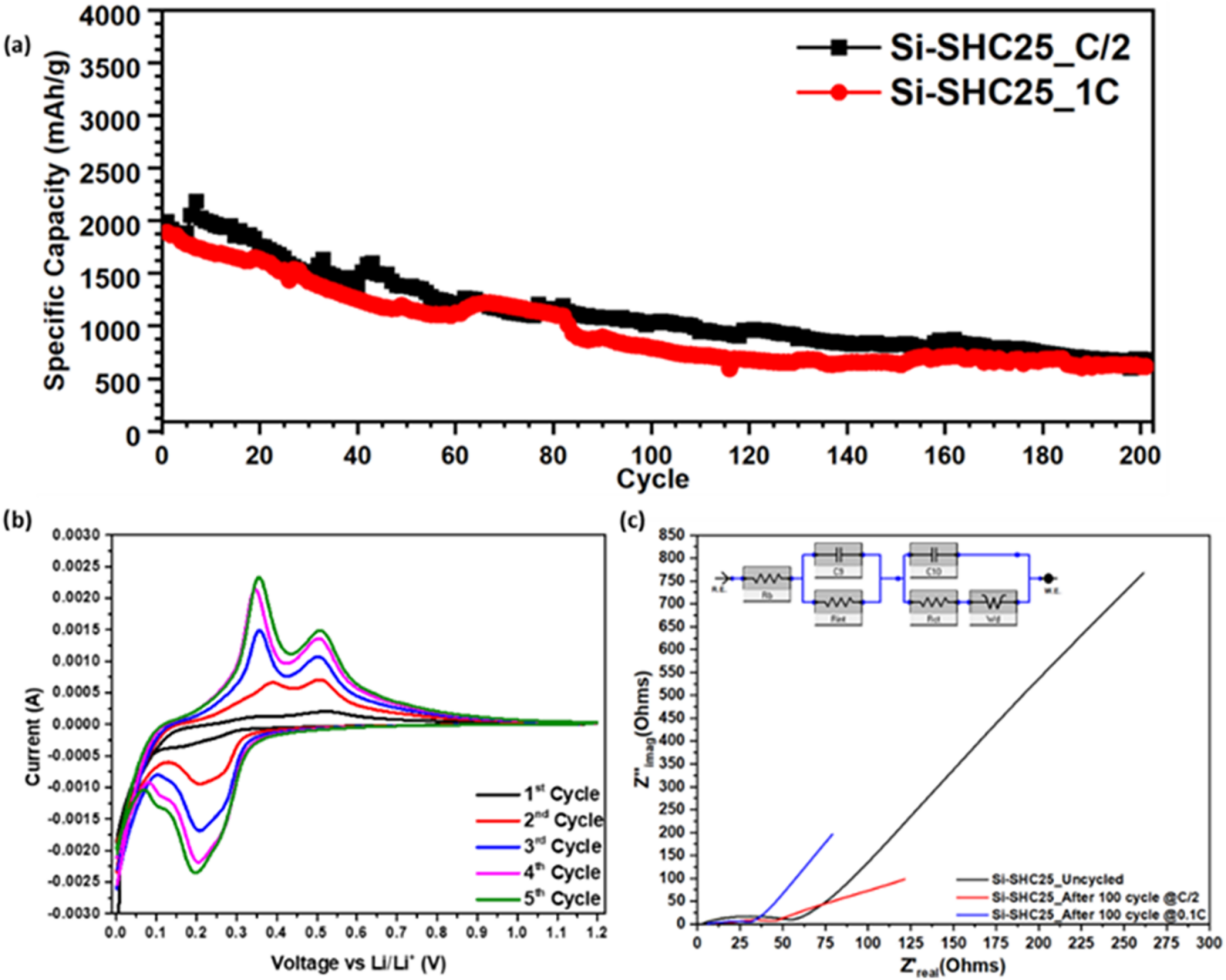
(a) Cycling test of Si-SHC25 at different C rates, (b) CV measurements of Si-SHC25, and (c) comparison of EIS results of the cycled and uncycled Si-SHC25 electrodes.

Therefore, electrode integrity can be more important than the specific capacity, especially in the case of the Si anode, because of the volume expansion of Si active particles and electrode fragmentation during the galvanostatic charge/discharge process. Cyclic voltammetry measurements were applied to the Si-SHP25 half-cell at a 0.1 mV constant voltage. The Si and SiO_*x*_ regions of the Si-SHC25 electrode oxidation peaks can be clearly observed at 0.357 and 0.508 V in the third cycle, respectively (Figure 7b). The Si oxidation peak shifts to 0.344 V in the fourth cycle during the CV measurement, and the shifted oxidation peak replaces the third cycle in the fifth cycle (Figure 7b). The impedances of the uncycled electrode, the 100-cycled electrode at C/10, and the 100-cycled electrode at C/2 are compared in Figure 7c. It was found that the uncycled electrode showed 56 ohm impedance (*R*_ct_). However, cycled Si-SHC25 electrodes even showed volume changes and a lower charge transfer resistance after cycling, demonstrating the electrode integrity with the conductive and self-healing behavior of the SHC polymer.

PANI in the self-healing polymer becomes more active when current is applied due to the electron–hole characteristics of the conductive polymer during the cycle.^73^ Si-SHC25 at C/10 showed 30 ohm impedance, and Si-SHC25 at C/2 showed 43 ohm impedance after 100 cycles.

SEM investigation of the Si-SHC25 electrode was performed to study the interaction of the Si-SHC binder and self-healing mechanism. The Si-SHC25 electrode showed a homogeneous and uniform surface with a grainy appearance, as observed in the SEM image in Figure 8a. After the cycling test at C/2, the Si-SHC25 half-cell was disassembled, and SEM images of the cycled electrodes were acquired as proof of the healed part of the electrode.

**Figure 8:**
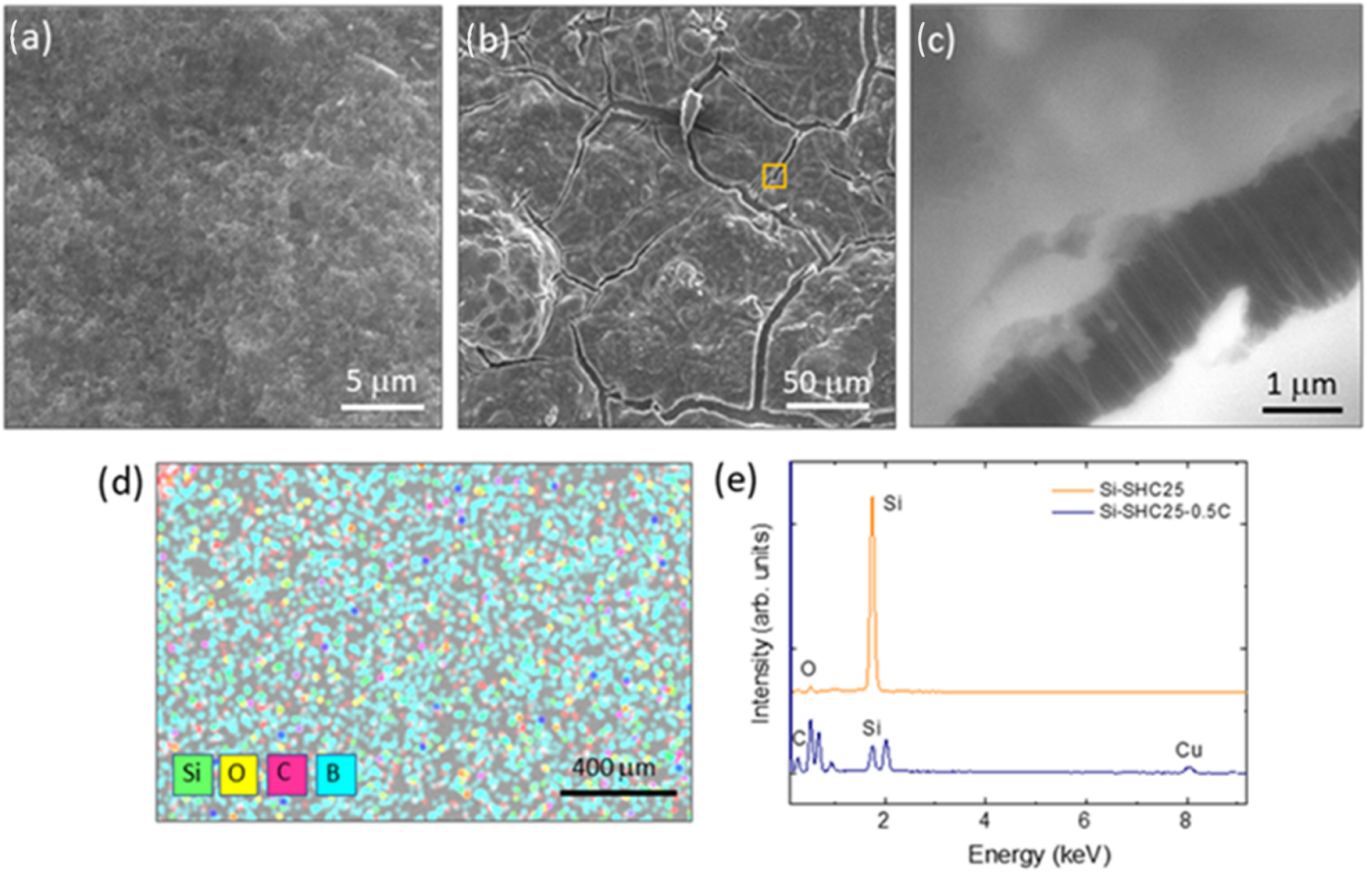
SEM images of the Si-SHC25 electrode (a) before cycling, (b) after cycling, (c) showing the self-healing bond region marked in (b). (d) EDS mapping of the electrode Si-SHC25 before cycling, and (e) EDS spectra of the Si-SHC25 electrode before and after cycling.

In this case, microcracks and inhomogeneous views appeared at the surface of the electrode after cycling, as shown in Figure 8b. A detailed analysis confirmed that small nanofibers/wires, 1–2 μm in length and 10–30 nm in width, could be observed in the cracks appearing in the cycled electrode (Figure 8c). These nanofibers tend to inhibit the separation of the regions on both sides of the cracks, hence retaining the surface uniformity during cycling. These fibers, as fingerprints of the self-healing mechanism, mainly appear in cracks with lower dimensions. A detailed analysis of crack surfaces revealed the presence of nanofibers/wires with diameters ranging from 10 to 30 nm within all observed cracks. In contrast, in the SEM image shown in Figure S10, no such nanostructures were detected in the cracks of the cycled anode without the self-healing polymer (Si/PVDF at the 30th cycle), indicating that the formation of these nanofibers/wires was closely associated with the presence of the self-healing component.

Homogeneous dispersion of the SHC polymer and Si was observed in the electrodes before cycling, as confirmed by EDS mapping in Figure 8d. It is easily seen that uniform Si, C, and B element dispersion was obtained in the EDS analysis, where the B element comes from 3-aminophenylboronic acid in the poly­(aniline-*co*-3-aminophenylboronic acid)/PVA binder. Although the SHC polymer maintained the integrity of the electrode with its self-healing feature, the fast charge/discharge of Si-SHC25 caused some deformation of the anode. It is known that during the charge/discharge process, HF and other side products, such as LiOH, Li_2_CO_3_, LiF, ROLi, etc., are revealed due to electrolyte decomposition.^74^

In the latest research demonstrated by Kim et al., the HF interaction with the Si anode causes Si active particles to corrode and react with oxides, resulting in the SiO_*x*_ species.^65^ Figure 8e shows the EDS spectra acquired for the Si-SHC25 electrode before and after cycling.^73^ In the initial electrode, Si is the main element, while after cycling, a significant decrease in the Si composition is observed, due to pulverization effects, together with an increase of other elements, such as O and C, owing to oxidation and pulverization effects. Actually, the Si:O ratio in the electrode changed from 4.3 before cycling to 0.1 after cycling, according to the elemental quantification of the EDS spectra. The increase in the Si:O ratio is related to the reaction of the Si active materials with byproducts and Si dissolution on the Si-SHC25 electrode during the charge/discharge process.

## 3 Conclusions

Si elements are the most promising elements for storing Li^+^ ions in LIBs. However, dramatic volume changes in the Si element during charging cause rapid capacity reduction. Therefore, Si-based anodes should overcome this capacity reduction caused by volume changes. Smart materials such as self-healing polymers compensate for the volume change due to capacity reduction. We synthesized a poly­(aniline-*co*-3-aminophenylboronic acid)/PVA (SHC) self-healing binder and integrated it into a Si anode electrode with a PVP colinker. Aniline fingerprint peaks and hydrogen bond peaks of SHC were observed by FTIR spectroscopy. In addition, the decomposition temperature of SHC was determined by DTGA. SHC’s self-healing ability was verified by the handmade cut-and-paste method. All parameters confirmed the successful synthesis of self-healing poly­(aniline-*co*-3-aminophenylboronic acid)/PVA. Electrochemical analysis showed that the SHC binder greatly improved the Si anode capacity. The Si-SHC25 electrode showed a delithiation capacity of 4125 mAh/g, which was 3 times higher compared to that of the Si-PVDF electrode. Although high C rates accelerated the degradation of the electrodes, the SHC binder worked successfully on the Si electrodes. Si anode worked 200 cycles at high C rates with the help of the SHC binder. Post-mortem analysis of the cycled Si anode electrode clearly demonstrated the self-healing bond of the SHC using SEM. The SHC polymer acted as a self-healing binder with a finely stretched polymer structure capable of holding the active material together on the Cu foil surface, as shown in the SEM images. As a result, although some cracks were formed during charge/discharge, poly­(aniline-*co*-3-aminophenylboronic acid)/PVA polymer successfully integrated into the Si-based anode material to improve the capacity.

## Supplementary Material



## Notes

Experimental section, including materials, synthesis, characterization techniques, and cell production (PDF)
