# Supplementary material for: Multifunctionalized Conductive Polymers for Self-Healing Silicon Anodes in Li-Ion Batteries
Source: ACS Omega. 2025 Jul 24;10(30):33607–18. doi: 10.1021/acsomega.5c04052 (PMC12332679; doi:10.1021/acsomega.5c04052)
Supplement: Supplementary file 1 [file ao5c04052_si_001.pdf]

SUPPORTING INFORMATION

Multifunctionalized Conductive Polymer for Self-Healable Silicon Anodes in Li-ion Batteries

Neslihan Yuca[1, 2*], Omer Suat Taskin[1, 3*], Emre Guney[1], Javier García-Alonso[4], David Maestre[4], Bianchi Méndez[4]

[1] *Enwair Energy Technologies Corporation, Kağıthane, Istanbul 34415, Turkey*
[2] *Institute of Energy, Istanbul Technical University, Istanbul 34469, Turkey*
[3] *Department of Chemical Oceanography, Institute of Marine Science and Management, Istanbul University, Istanbul 34134, Turkey*
[4] *Departamento de Física de Materiales, Facultad de CC. Físicas, Universidad Complutense de Madrid, Madrid, 28040, Spain*

Materials

3-aminophenylboronic acid, PVA (Mw: 30000-50000 g/mol), Aniline hydrochloride, Ammonium persulfate (APS), %37 HCl were purchased from Sigma-Aldrich and used as received. PVDF(HSV900), Conductive carbon (TIMICAL SUPER C65) were obtained from MTI. CMC (A18105) were obtained from Alfa-Aesar and SBR from Nanografi. Tuball (TUBALL BATT H_2O 0.2% PVP) were obtained from OCSiAl company.

Synthesis of poly(aniline-*co*-3-aminophenylboronic acid)/PVA

3-aminophenylboronic acid was dissolved in appropriate amount of water. After the dissolution, at ambient temperature, the solution was cooled down to 0 °C inside an ice bath. The PVA was dissolved in an appropriate amount of water and mixed with aniline hydrochloride at ambient temperature. The solution was cooled down to 0 °C and was added to the 3-aminophenylboronic acid solution. Finally, APS was added to the mixed solution to initiate polymerization. Transparent aniline hydrochloride was mixed with the PVA solution (Fig. S1a). After the polymerization, aniline hydrochloride and 3-aminophenylboronic acid were reacted to form poly(aniline-*co*-3-aminophenylboronic acid)/PVA (SHC) with emeraldine salt structure (Fig. S1b). The synthesized composite product (SHC) was obtained in the form of a dark green hydrogel and the yield was 84% (Fig. S1c). The resulting hydrogel SHC was dried in a vacuum oven at 60 °C and then ground in a ball mill at 1000 rpm for 1 h to prepare the SHC integrated Si anode (Fig. S1d).

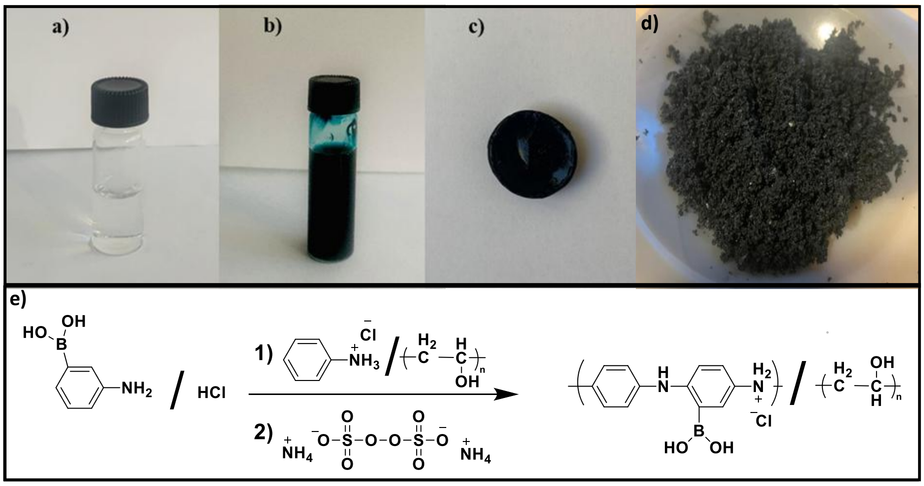

Figure S1. a) Aniline Hydrochloride solution, b) Polymerization media of 3-boronic acid aniline& aniline, c) Obtained poly(aniline-*co*-3-aminophenylboronic acid)/PVA composite binder, d) Dried and granulated SHC binder, e) Synthesis mechanism of SHC binder.

In order to understand the morphology of the composite, SEM measurements have been provided. SEM images, which are seen in Figure S2, show various morphology with structures usually bellow 10 µm and variable sizes. Some parts of the polymer composite presenting a higher secondary electron yield than others which might suggest compositional variations. It is clear that high magnification observation suggests a laminar structure at the structure surface.

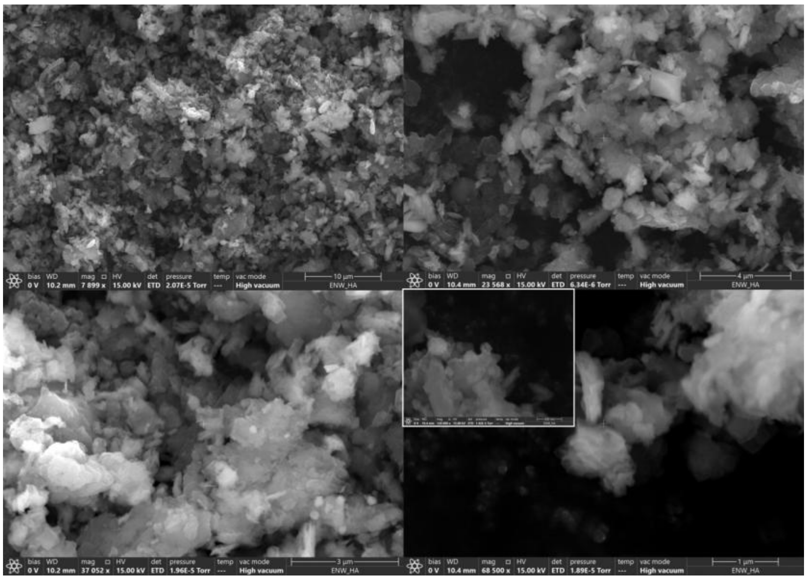

Figure S2. SEM images of the poly(aniline-*co*-3-aminophenylboronic acid)/PVA composite in different magnifications

Raman spectrum have been performed to support FTIR analysis for detail investigation of the structure (Figure S3). The Raman spectrum of poly(aniline-co-3-aminophenylboronic acid)/PVA composite shows obvious characteristic points reflecting the structure of the copolymer and PVA matrix. The peak at about 1607 cm^{-1} corresponds to the C=C stretching vibration in the quinoid ring of the polyaniline backbone. The expected carbon modes D and G appear at the sample at 1357 and 1607 cm^{-1}.

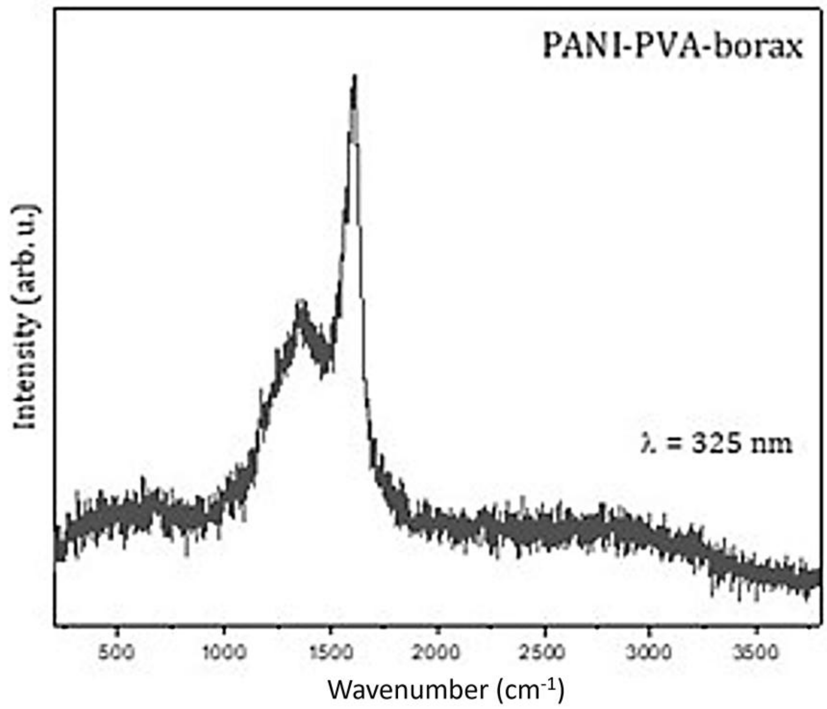

Figure S3. Raman spectrum of SEM images of the poly(aniline-co-3-aminophenylboronic acid)/PVA composite

Cell production

Si-SHC-10 and Si-SHC-25 anode electrodes (7:1:1.2:0.8 and 5.5:2.5:1.2:0.8 of ratios) were prepared with pure Silicon, SHC, Carbon nanotubes (CNTs) and PVP co-binders, respectively. Tuball was used for PVP co-binder and CNTs solution. First calculated amount of Tuball® was added to the 50 mL beaker. Then SHC binder was added to the co-binder-CNTs solution and stirring half an hour. After stirring, Pure Si was added to the obtained homogeneous solution and continued stirring until pure homogenous green slurry was obtained. Slurry was coated with 150 µm thickness doctor blade on a copper foil and dried at 80 °C in the bake oven. The same preparation method was applied to Si-PVDF [1] (8:1:1 of ratio) and Si-CMC-SBR (7:1:1:1 of ratio) [2] anode electrodes for comparison. PVDF and CMC-SBR binder systems have been used as commercial polymers in the industry since the beginning of Li-ion battery production. Therefore Si-PVDF and Si-CMC-SBR electrodes were prepared as comparison electrodes. The areal mass loading is on average 0.8 mg/cm^2 (based on Si). The electrodes were cut with a coin cell cutting apparatus with a disk area of 1.8 cm^2. The coin cell sized electrodes were taken into the glovebox and assembled in a 2032 coin cell case as a half-cell with Li metal as counter electrode and 1M $LiPF_6$ EC:DEC (1:1) electrolyte in argon atmosphere. Electrode capacities were calculated by multiplying the Si weight of the disk-shaped electrodes by the

theoretical capacity of the Si active material, which was considered as 3500 mAh/g. The obtained values were considered as 1C, and electrochemical analyses, as described in the article, were measured according to 1C derivatives, and the galvanostatic charge-discharge tests were performed between 0.01V-1.2V in the half-cell configuration where Li metal was used as a counter electrode.

Material characterization

The experimental procedure for acquiring FTIR spectra before and after healing begins with sample preparation, where a polymer film (2 cm x 4 cm) is fabricated through polymerization in a vial. A controlled damage (cut) is introduced to the film prior to the initial FTIR measurement, which is performed in ATR mode (wavenumber range: 4000–400 cm^{-1}, resolution: 4 cm^{-1}, 16 scans), with a background scan collected from air as reference. After the pre-healing measurement, the sample undergoes healing via autonomous recovery, depending on the material's healing mechanism through H bonding. Post-healing, the same damaged region is re-analyzed under identical FTIR conditions to track changes in functional groups. Peak intensity and shift comparisons before and after healing reveal molecular-level recovery, with replicates and environmental controls applied as needed to ensure reproducibility.

Differential Thermal Gravimetric Analysis (DTGA) analysis was measured with Shimadzu DTG-60H Differential Thermal Gravimetric Analyzer. FT-IR analysis was carried out with Perkin Elmer Spectrum Two-FTIR system. Morphological analysis was performed in a FEI-Inspect S50 scanning electron microscope (SEM) and a Hitachi TM3000 SEM using acceleration voltages of 10–15 kV. Compositional analysis was also performed by energy dispersive X-ray spectroscopy (EDS) in Hitachi TM3000 SEM equipped with a Quantax70 detector, using an acceleration voltage of 15 kV. Electrochemical impedance spectroscopy and cyclic voltammetry were measured using a Gamry reference 3000 Potentiostat/Galvanostat. Cycling test was performed in a Neware battery testing system.

The conductivity property of the SHC binder was measured by DC impedance measurement using the Hioki IM3536 LCR meter. The sample was pressed with 13 mm diameter and was sandwiched between the gold plated and stainless steel. The sandwiched SHC sample was placed under Novocontrol BDS 1200 sample holder and conductivities were measured at different temperature ranges (20-90 °C in 10 °C intervals) in the DC mode of the LCR meter. The DC conductivity property of the SHC pellet was measured using equation 1;

$$\sigma_{dc} = l /(R_{dc} * A) \quad (1)$$

Where σ_{dc} is the DC conductivity, l is the thickness of the SHC (cm), R_{dc} is the measured resistance (Ω) and A is the area (1.33 cm^2) of the SHC pellet.

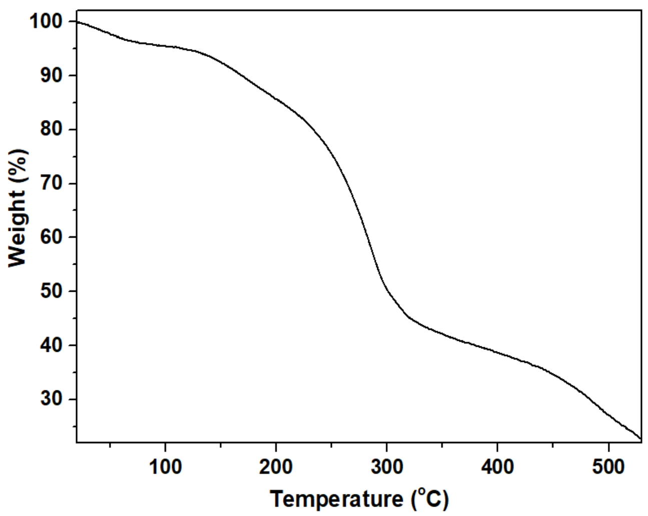

Figure S4. TGA profile of PANI-*co*-PANI (boronic acid)/PVA

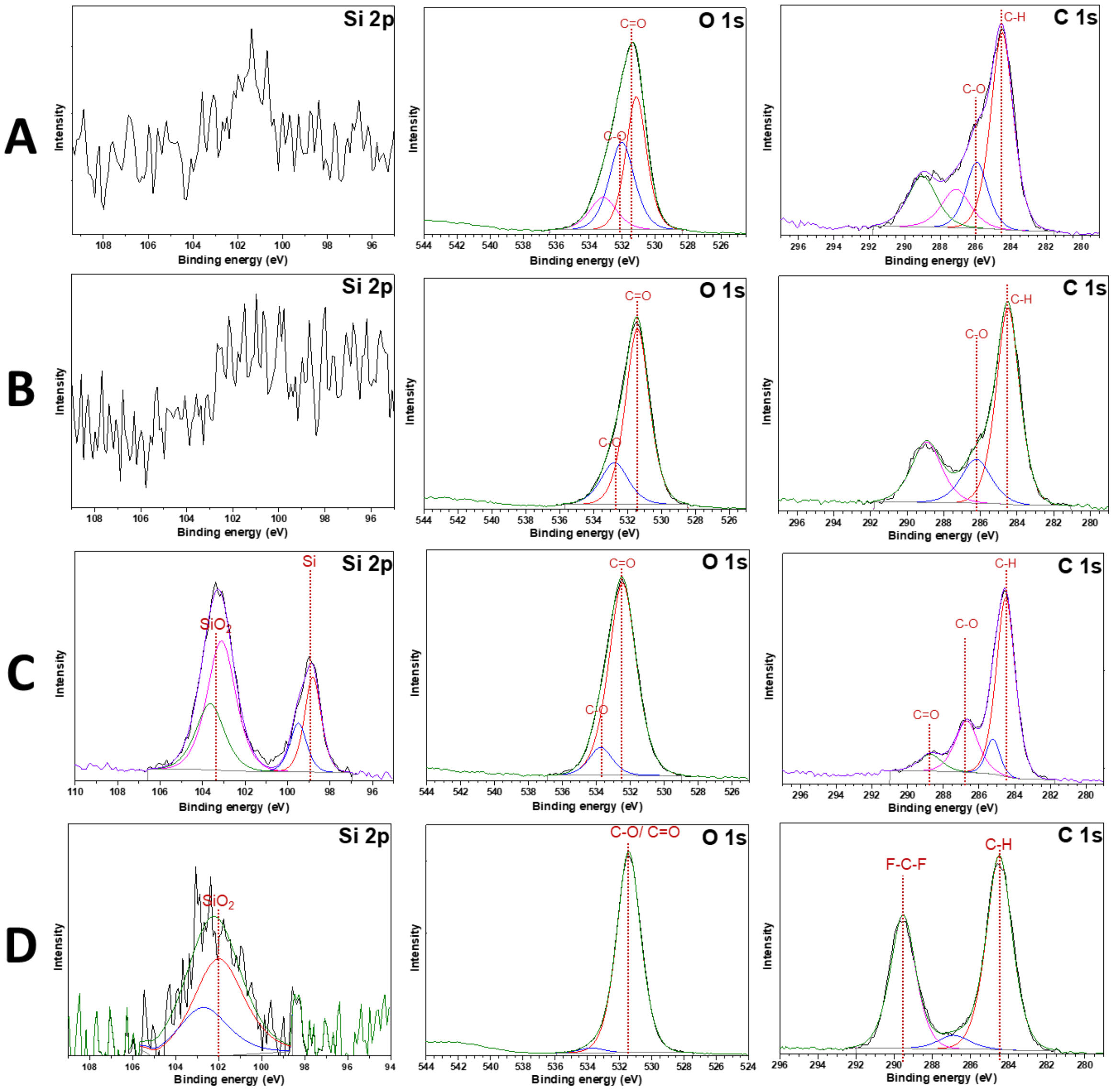

Figure S5. XPS measurements of cycled A) Si-SHC25 electrode, B) Si-SHC10 electrode C) Si-CMC/SBR electrode and D) Si-PVDF electrode.

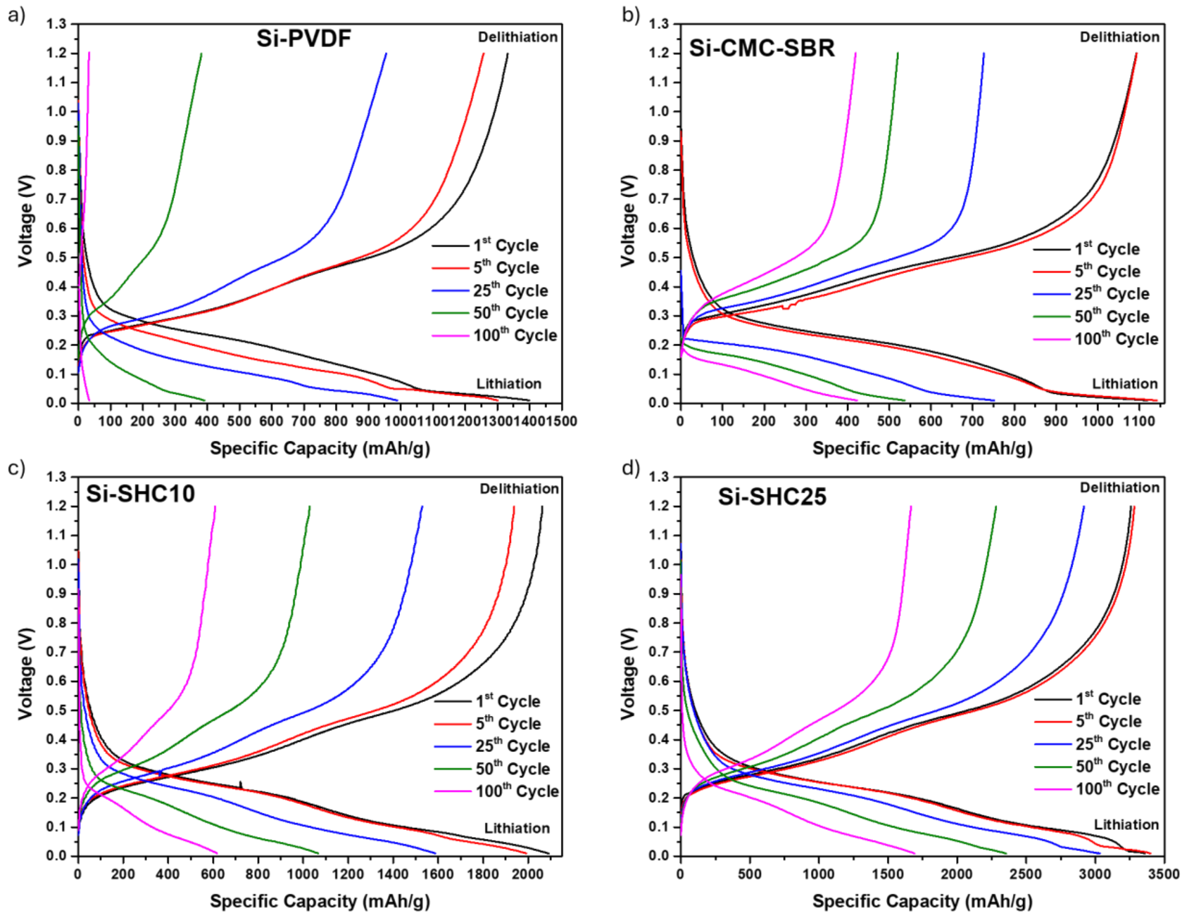

Figure S6. Capacity-Voltage curves of the a) Si-PVDF, b) Si-CMC-SBR, c)Si-SHC10, d) Si-SHC25 electrodes during the GCD long-term cycling test.

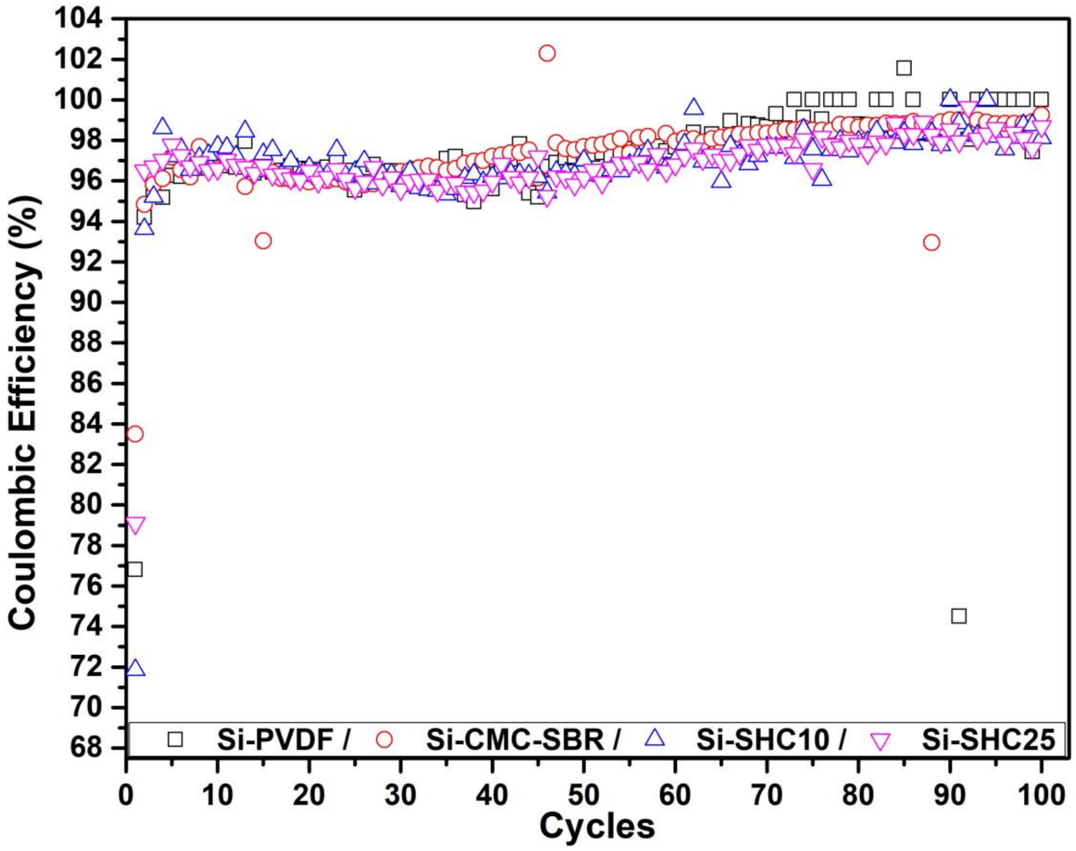

Figure S7. Coulombic efficiency of the electrode during the galvanostatic charge-discharge cycles.

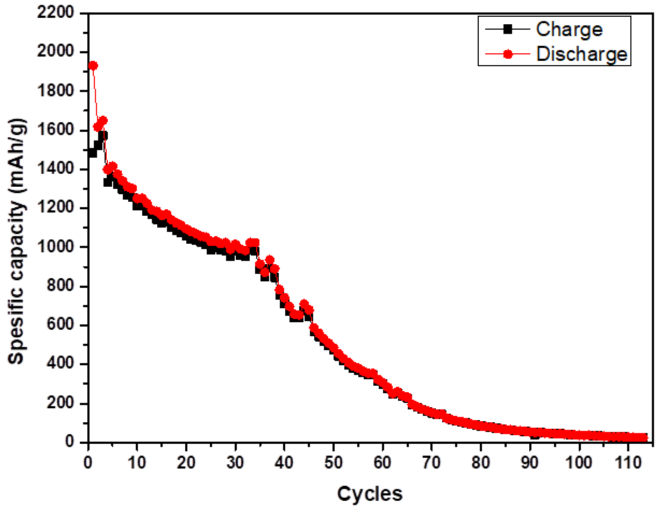

Figure S8. Galvanostatic charge-discharge result of Si-PVDF electrode at C/2.

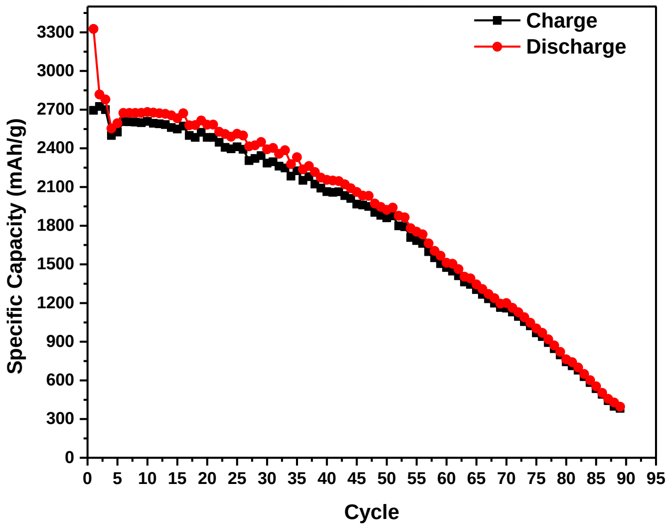

Figure S9. Galvanostatic charge-discharge result of Si-CMC/SBR electrode at C/2.

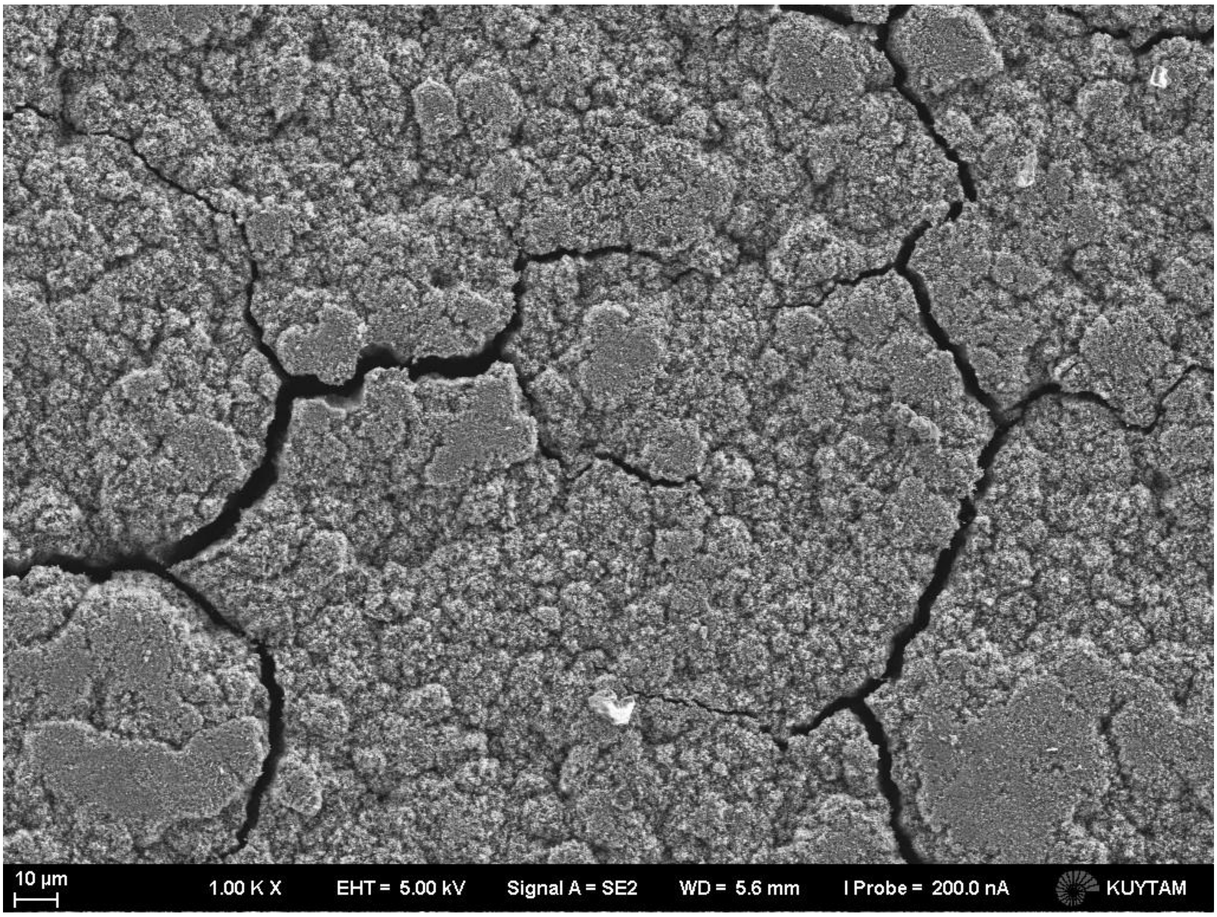

Figure S10. SEM image of cycled Si/PVDF anode

References

(1) Huang, H., Han, G., Xie, J., & Zhang, Q. The effect of commercialized binders on silicon oxide anode material for high capacity lithium ion batteries. Int. J. Electrochem. Sci, 2016, 11(10), 8697-8708.

(2) Tzeng, Y., Jhan, C. Y., Wu, Y. C., Chen, G. Y., Chiu, K. M., & Guu, S. Y. E. High-ICE and high-capacity retention silicon-based anode for lithium-ion battery. Nanomaterials, 2022, 12(9), 1387.
